# Influence of constraint mechanism on kinematics, contact mechanics, and polyethylene wear in dual-axis rotating hinge and ball-and-socket knee prostheses: a finite element study

**DOI:** 10.3389/fbioe.2026.1942105

**Published:** 2026-09-23

**Authors:** Jinwei Liu, Xiaoyi Wang, Chuanhao Guo, Yongcheng Hu, Weiguo Xu, Jingyu Zhang

**Affiliations:** 1 Department of Bone Tumor and Soft Tissue Oncology, Tianjin Hospital, Tianjin University, Tianjin, China; 2 Clinical School, College of Orthopaedics, Tianjin Medical University, Tianjin, China

**Keywords:** finite element model, kinematics, prosthesis design, rotating hinge knee, wear simulation

## Abstract

**Background:**

Severe metaphyseal bone defects following revision total knee arthroplasty or tumor resection often require highly constrained knee prostheses. Although rotating hinge knee (RHK) designs provide reliable stability, polyethylene wear and aseptic loosening remain important concerns. A novel ball-and-socket knee prosthesis has been developed to improve rotational accommodation and joint kinematics; however, its biomechanical performance relative to conventional rotating hinge designs remains unclear.

**Methods:**

Finite element models of a dual-axis rotating hinge knee prosthesis and a ball-and-socket knee prosthesis were developed and subjected to five million gait cycles under ISO 14243-1:2009 loading and motion conditions. Kinematics, contact mechanics, and polyethylene wear were evaluated using a contact-pressure-independent modified Archard wear formulation incorporating cross-shear effects. The reported wear volume for the ball-and-socket prosthesis represents the analyzed bearing side and its associated constraint interfaces.

**Results:**

Both prosthetic designs exhibited limited anterior–posterior translation and maintained relatively low contact pressures due to highly conforming articulations. Compared with the dual-axis rotating hinge prosthesis, the ball-and-socket prosthesis demonstrated greater internal–external rotational freedom (21.6° vs. 3.2°) and broader contact migration during gait. Although the ball-and-socket design exhibited larger contact areas and lower contact pressures, its polyethylene wear rate (10.06 mm^3^/million cycles) was higher than that of the dual-axis prosthesis (1.78 mm^3^/million cycles). This difference corresponded to the greater multidirectional motion and cross-shear conditions observed in the ball-and-socket design.

**Conclusion:**

Prosthetic design influences kinematics, contact mechanics, and wear behavior in highly constrained knee systems. The results indicate that multidirectional motion and cross-shear may play a more important role in polyethylene wear than contact pressure alone. Although the ball-and-socket prosthesis exhibited greater wear than the dual-axis design, it provided increased rotational accommodation and may better accommodate rotational demands during gait. These features support its potential as an alternative design strategy for constrained knee reconstruction after further experimental validation and design optimization. Future optimization should focus on reducing excessive multidirectional sliding and cross-shear while preserving rotational adaptability.

## 1 Introduction

Severe bone defects frequently occur following revision total knee arthroplasty or limb-salvage surgery after bone tumor resection. Management of these conditions is challenging because of extensive bone loss, soft-tissue insufficiency, compromised bone quality, and the absence of reliable anatomical landmarks. In these situations, highly constrained knee prostheses are often required to restore joint stability and function (Haijie et al., 2018; Kouk et al., 2018; Bingham et al., 2019; Neuhaus and Maier, 2022).

Early-generation hinged knee prostheses were associated with high failure rates due to excessive constraint, limited kinematic freedom, and inadequate material performance. Advances in implant design have led to the development of modern rotating hinge knee (RHK) systems incorporating rotating platforms, improved tibiofemoral conformity, highly cross-linked polyethylene bearings, and modular reconstruction components (Abdelgaied et al., 2011; 2018; Bingham et al., 2019; Arnholdt et al., 2020; Theil et al., 2020; Oliver et al., 2025). These developments have improved implant stability and mobility while reducing excessive constraint at the implant interfaces. Nevertheless, long-term survivorship of RHK prostheses remains lower than that reported for primary total knee arthroplasty, with 10-year survival rates ranging from 51% to 92.5% (Arnholdt et al., 2020; Petershofer and Trieb, 2020; León-Román et al., 2021). Aseptic loosening remains a major cause of failure. Although polyethylene wear and wear debris–induced osteolysis contribute to implant failure, prosthetic kinematics, multidirectional motion, and constraint transfer may also influence long-term implant performance in highly constrained knee systems (Billi et al., 2010; Reinders et al., 2015; Samiezadeh et al., 2019; Zhang et al., 2020; Levent et al., 2021; Oliver et al., 2025).

To address some of the limitations of conventional hinge designs, our group developed a ball-and-socket rotating hinge knee prosthesis in which a spherical connector integrates flexion-extension and internal-external rotational axes and allows multiplanar rotational accommodation while maintaining femorotibial load transfer. Previous studies demonstrated that this design achieves low contact pressures and allows greater rotational freedom under static loading conditions (Bus et al., 2017; Zhang et al., 2020). Polyethylene wear in less constrained knee prostheses, including cruciate-retaining and posterior-stabilized designs, has been extensively investigated using retrieval analyses, simulator studies, and computational models (Fregly et al., 2005; McEwen et al., 2005; Utzschneider et al., 2009; Billi et al., 2010). In contrast, studies focusing on highly constrained knee prostheses remain limited. Prediction of the wear performance of knee prostheses using numerical models has not been investigated for hinge knee prostheses. Whether the cross-shear-based wear prediction model is applicable to highly constrained hinge joints remains unclear. In particular, no studies have directly compared how different constraint mechanisms influence kinematics, contact mechanics, and polyethylene wear in highly constrained knee prostheses under identical loading conditions.

Therefore, the aim of this study was to develop finite element models of a dual-axis rotating hinge knee prosthesis and a ball-and-socket knee prosthesis to compare their kinematic behavior, contact mechanics, and polyethylene wear characteristics. We hypothesized that the ball-and-socket prosthesis would provide greater rotational accommodation than the dual-axis rotating hinge design, but that the resulting multidirectional motion and contact migration would produce distinct polyethylene wear behavior.

## 2 Methods

### 2.1 Construction of finite element models of dual-axis and ball-and-socket prostheses

Computer-aided design (CAD) models of two highly constrained knee prosthesis systems were evaluated in this study: a dual-axis rotating hinge knee prosthesis and a ball-and-socket rotating hinge knee prosthesis (Beijing Weigao Company, China), both previously developed by our research group (Zhang et al., 2020) (Figure 1).

**FIGURE 1:**
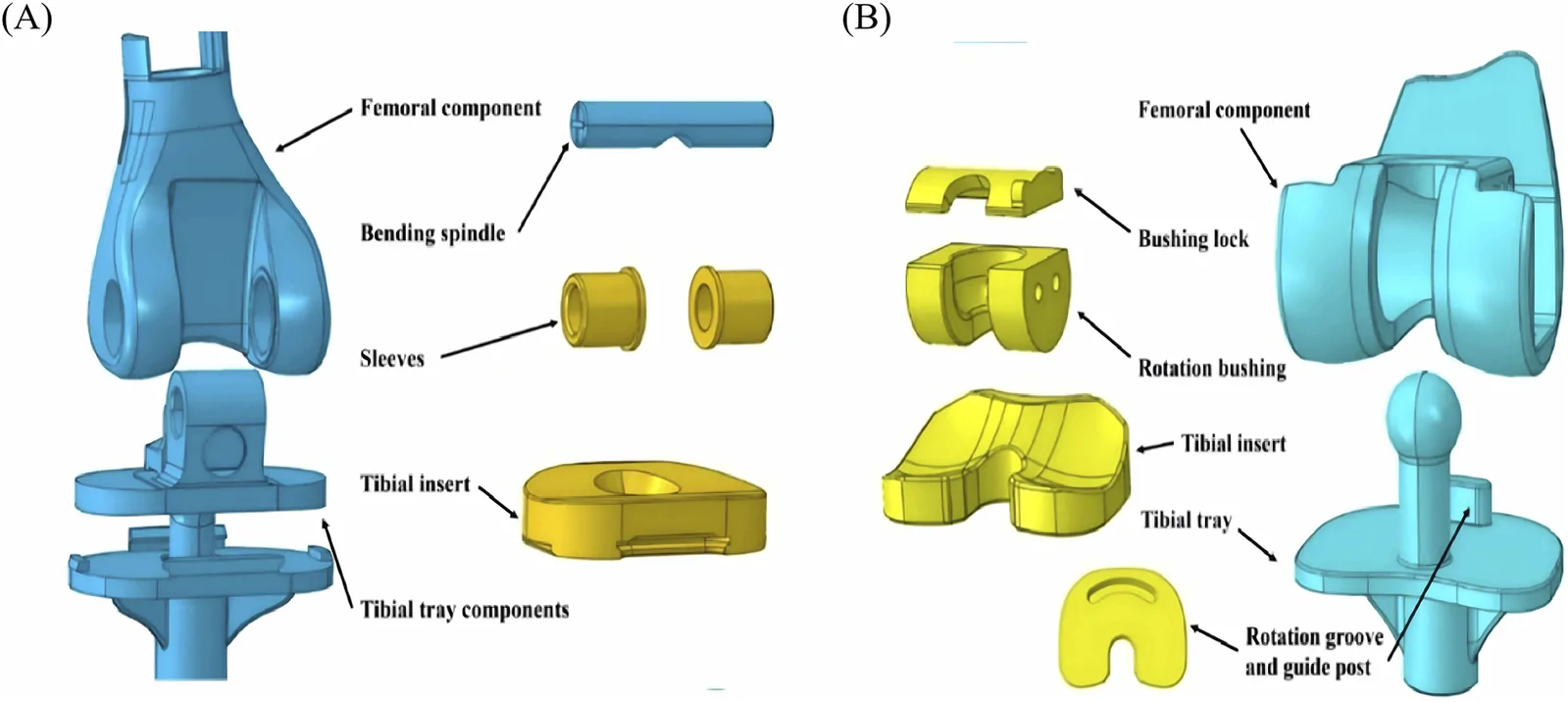
The CAD model and component parts of the two prostheses: **(A)** dual-axis prosthesis; **(B)** ball-and-socket prosthesis.

The dual-axis rotating hinge prosthesis consisted of two polyethylene sleeves, a tibial insert, femoral components, a bending spindle, and a tibial tray assembly. The polyethylene insert was rigidly fixed to the tibial tray (Figure 1A). Flexion–extension was accommodated through the bending spindle, whereas internal–external rotation occurred between the tibial tray and the longitudinal connecting axis. This design constrains rotational motion to a predefined axis and provides a relatively constrained kinematic mechanism.

The ball-and-socket knee prosthesis consisted of a rotating bushing, a bushing lock, a polyethylene tibial insert, femoral components, and a tibial tray (Figure 1B). The femoral and tibial components were connected through a spherical column-and-socket mechanism, allowing multi-axial motion while maintaining load transfer through the femoral condyles. Compared with the dual-axis design, this configuration permits greater rotational accommodation and a less constrained kinematic pattern. The CAD geometries were reconstructed directly from the original implant designs without geometric simplification.

### 2.2 Finite element model

Finite element models of the two prosthetic systems were developed from the corresponding CAD geometries and implemented in ABAQUS 6.18 (Simulia, Providence, RI, USA). The models were constructed to evaluate kinematics, contact mechanics, and polyethylene wear under dynamic loading conditions.

Boundary conditions were defined according to ISO 14243-1:2009 and included axial load, flexion–extension (FE) rotation, anterior–posterior (AP) force, and internal–external (IE) rotational torque. In the absence of dedicated testing standards for rotating hinge knee prostheses, ISO 14243-1:2009 was adopted to provide consistent loading conditions for comparison between the two prosthetic designs. Positive AP motion corresponded to anterior translation, whereas positive IE rotation represented internal rotation.

#### 2.2.1 Dual-axis rotating hinge prosthesis

For the dual-axis prosthesis (Figure 2A), axial load was applied to a reference node on the femoral component located along the bending spindle axis and offset medially by 5 mm. Flexion-extension motion was prescribed at the femoral component, whereas adduction-abduction was left unconstrained. The remaining femoral degrees of freedom were constrained (Table 1). The polyethylene insert was rigidly attached to the tibial tray; therefore, backside wear of the insert was not considered. AP force and IE rotational torque were applied to the reference node of the tibial tray along the longitudinal axis of the tibia, while the remaining degrees of freedom were constrained. The superior surface of the polyethylene insert articulated against the corresponding metallic femoral component.

**FIGURE 2:**
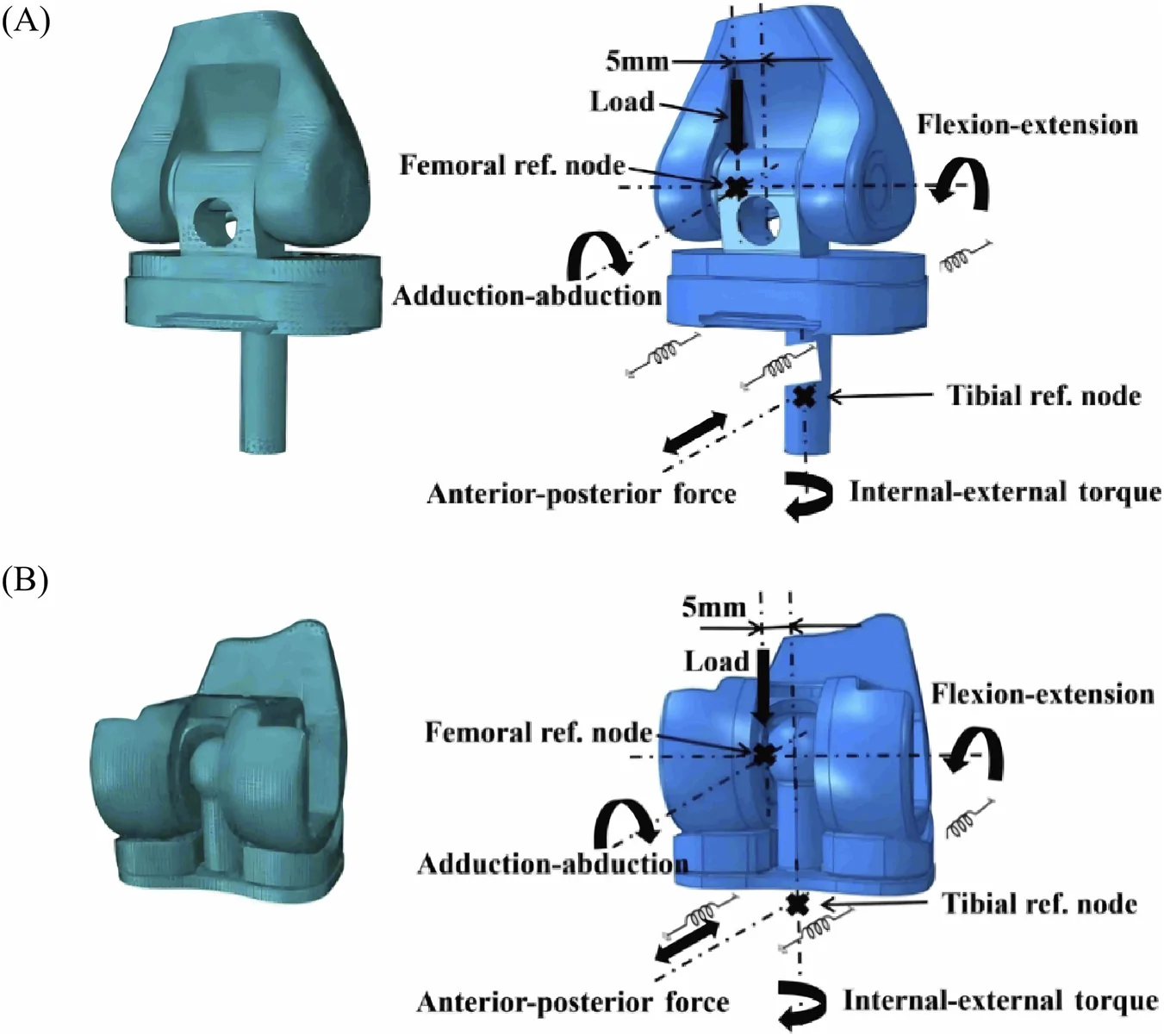
Illustration of FE models and motion of the two prostheses: **(A)** the dual-axis prosthesis; **(B)** the ball-and-socket prosthesis.

**TABLE 1:** Prescribed and constrained degrees of freedom for the dual-axis and ball-and-socket prostheses.

Prosthesis type	Degrees of freedom				
	FE	IE	AA	ML	AP
Dual-axis prosthesis	✓	✓	×	×	×
Ball-and-sock prosthesis	✓	✓	×	×	×

* FE, flexion-extension; IE, internal–external rotation; AA, abduction–adduction; ML, medial–lateral translation; AP, anterior–posterior translation.

#### 2.2.2 Ball-and-socket knee prosthesis

For the ball-and-socket prosthesis (Figure 2B), axial load and flexion-extension motion were applied to a reference node located at the center of the spherical connector and offset medially by 5 mm. Adduction-abduction of the femoral component was permitted, whereas the remaining degrees of freedom were constrained. For the ball-and-socket prosthesis, the articular surface of the polyethylene insert and the rotating bushing were included in the wear analysis because these surfaces served as functional bearing or constraint interfaces during gait. AP force and IE rotational torque were applied to the reference node of the tibial tray, whereas all degrees of freedom of the polyethylene insert were left unconstrained (Table 1). The AP translational spring stiffness and IE rotational resistance were set to 9.3 N/mm and 0.13 Nm/°, respectively, in accordance with ISO 14243-1:2009.

#### 2.2.3 Material properties and contact definitions

Ultra-high-molecular-weight polyethylene (UHMWPE) components, including inserts, sleeves, and bushings, were modeled as nonlinear isotropic elastic materials with an initial elastic modulus of 463 MPa and a Poisson’s ratio of 0.46 (Abdelgaied et al., 2011). The UHMWPE material was defined with its real stress-strain curve adopted, as shown in Figure 3 (Godest et al., 2002). All metallic components were modeled as rigid bodies because their deformation was negligible relative to that of the UHMWPE components. Tangential contact behavior was defined using a penalty-based friction formulation with a coefficient of friction of 0.04 (Willing and Kim, 2009). For the dual-axis prosthesis, contact interfaces included the sleeve-spindle interaction and the articulation between the superior surface of the polyethylene insert and the corresponding femoral component. For the ball-and-socket prosthesis, contact interfaces included the femoral component-polyethylene insert articulating surface, the insert backside-tibial tray interface, and the interaction between the rotating bushing/bushing lock and the spherical portion of the tibial tray.

**FIGURE 3:**
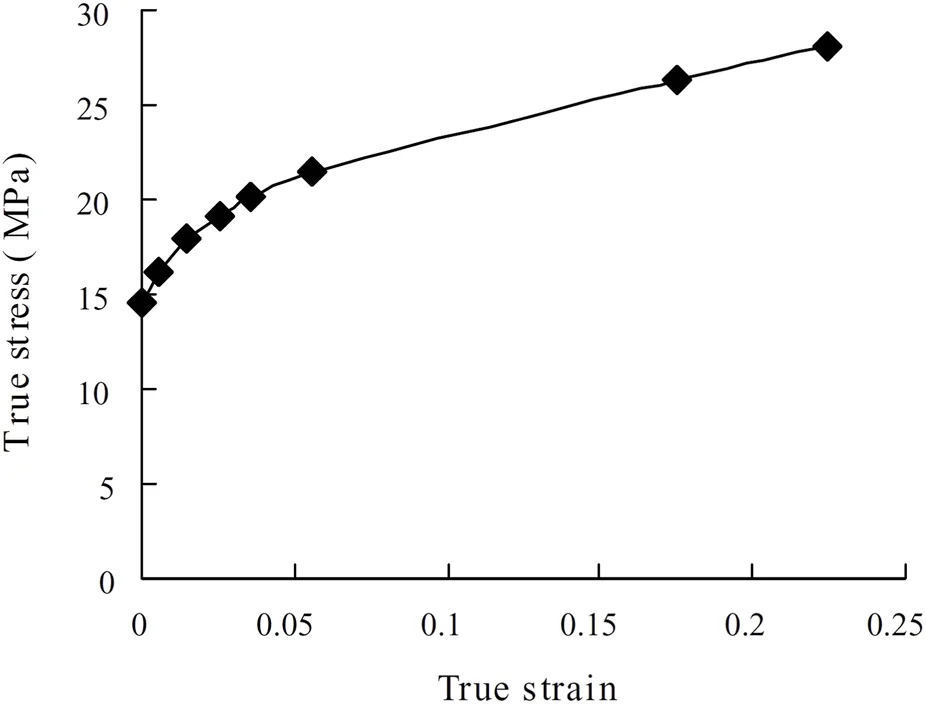
Real stress-strain curve of the UHMWPE.

Most components were meshed using quadratic tetrahedral elements (C3D10M), whereas the sleeves and spindle of the dual-axis prosthesis were meshed using linear hexahedral elements (C3D8I). Mesh convergence was assessed through progressive mesh refinement. A mesh was considered converged when further refinement resulted in less than 5% variation in peak contact pressure under the maximum axial load. Based on this criterion, a global element size of approximately 1–1.5 mm was selected for all simulations. The ball-and-socket insert and rotating bushing contained 167,784 and 58,953 elements, respectively, whereas the dual-axis insert and sleeves contained 52,023 and 2,292 elements.

### 2.3 Wear simulation

Polyethylene wear in both prosthetic designs was predicted using a contact-pressure-independent modified Archard wear formulation incorporating cross-shear effects (Abdelgaied et al., 2011). In this formulation, volumetric wear was assumed to be proportional to the wear coefficient, contact area, and sliding distance and was calculated as:

V=C·A·S (1)

where V is the volumetric wear, C is the dimensionless wear coefficient, A is the contact area, and S is the sliding distance. Nodal linear wear depth was calculated at each contact node and then integrated over the analyzed contact surface to obtain volumetric wear.

The wear coefficient (C) was defined as a function of the cross-shear ratio (CS), which quantifies the degree of multidirectional motion experienced by UHMWPE. The relationship between the wear coefficient and cross-shear ratio was established according to previously published multidirectional wear experiments on UHMWPE, which can be described as:

$$C={\left(a+b\times CS\right)}^{-1/c}$$ (2)

where *a*, *b* and *c* are constants. The *CS* was defined on the unified theory of wear and work (Wang, 2001; Kang et al., 2008a; 2008b), as the component of the frictional work perpendicular to the principal molecular orientation direction (*E*_*cross-shear*_), divided by the total frictional work (*E*_*total*_):

$$CS=\frac{E_{cross-shear}}{E_{total}}$$ (3)

Cross-shear values were obtained from multidirectional pin-on-plate wear experiments conducted under bovine serum lubrication, representing boundary and mixed lubrication conditions relevant to *in vivo* joint environments (Kang et al., 2008b; 2009; Fisher et al., 2010).

Creep deformation of UHMWPE was incorporated into the wear simulation framework, based on the experimental data reported (Lee and Pienkowski, 1998) The creep was modeled as:

$$\delta_{creep}=\left[3.491\times 10^{-3}+7.966\times 10^{-4}\left(\log\left(t\right)-4\right)\right]\sigma_{av}h$$ (4)

where *t* is time (min), $\sigma_{av}$ is the average pressure (MPa), and *h* is the thickness (mm) of UHMWPE components.

The total nodal surface displacement ( $\delta_{total}$ ) was calculated as the sum of wear-induced material removal ( $\delta_{wear}$ ) and time-dependent creep deformation ( $\delta_{creep}$ ):

$$\delta_{total}=\delta_{wear}+\delta_{creep}$$ (5)

Together, Equations 1–5 define the wear and creep calculation framework used in the simulation. The surface geometry was updated after each wear iteration to account for progressive geometric changes during the simulation. The nodal coordinates of the worn surface were modified using the total nodal surface displacement along the normal direction of the wear surface. At each simulation time step, contact pressure and sliding distance were extracted from the finite element results. A Python script was used to extract these data from the ABAQUS output database (ODB), while MATLAB (R2019b, The MathWorks, Natick, MA, USA) was employed to calculate nodal wear and creep and generate the updated finite element geometry for the subsequent simulation step.

A total of five million gait cycles were simulated. During the first one million cycles, four evenly spaced wear update iterations were performed to capture the pronounced effect of creep deformation. A higher update frequency was adopted during the initial stage because creep deformation is known to occur predominantly during the early cycles of polyethylene loading. Subsequently, one wear update iteration was conducted for each additional one million cycles. This iteration scheme was adopted based on preliminary convergence testing and was considered sufficient to capture the geometric evolution of the polyethylene components due to wear and creep. For the ball-and-socket prosthesis, the reported total wear volume refers to the articular surface and its associated constraint interfaces. The overall wear simulation workflow is summarized in Figure 4.

**FIGURE 4:**
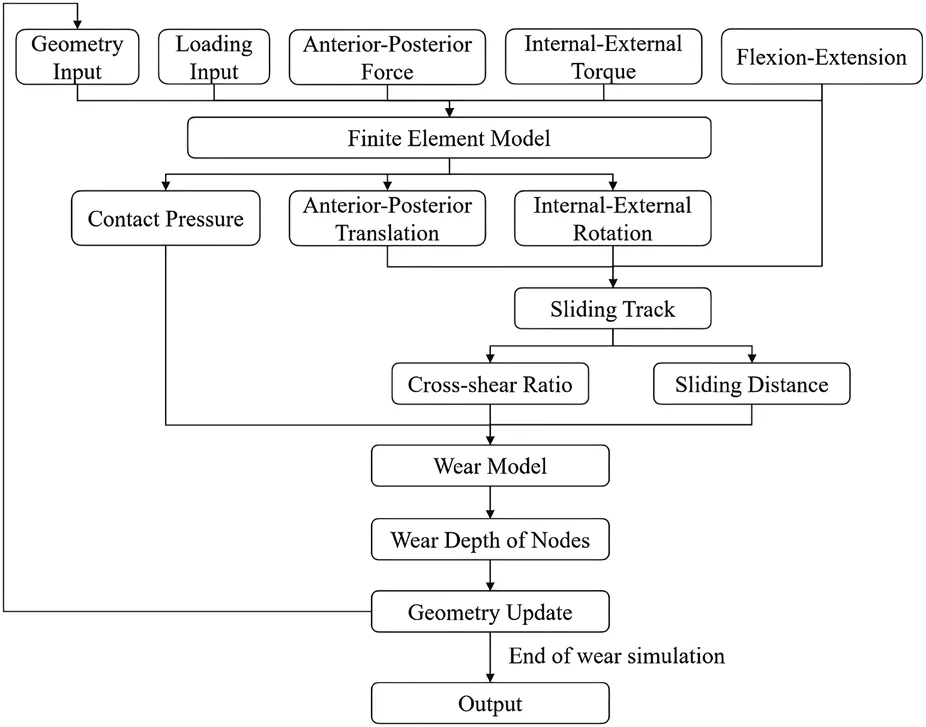
Flowchart for wear simulation of hinged knee prosthesis.

## 3 Results

### 3.1 Kinematics

The predicted anterior–posterior (AP) translation and internal–external (IE) rotation of the two prosthetic designs throughout the gait cycle are shown in Figure 5. For the dual-axis rotating hinge prosthesis, AP displacement of the tibial insert ranged from approximately −0.15 mm to 0.10 mm, with a peak-to-peak amplitude of 0.25 mm (Figure 5A). A small anterior translation peak occurred at approximately 20%–25% of the gait cycle, followed by gradual posterior translation reaching a maximum at approximately 60% of the gait cycle. Similar AP motion patterns were observed throughout the wear simulation. IE rotation remained limited throughout the gait cycle, with a peak-to-peak rotational excursion of approximately 3.2°. A transient internal-rotation peak occurred near 60% of the gait cycle (Figure 5B), and the overall rotational pattern showed no substantial change with increasing wear.

**FIGURE 5:**
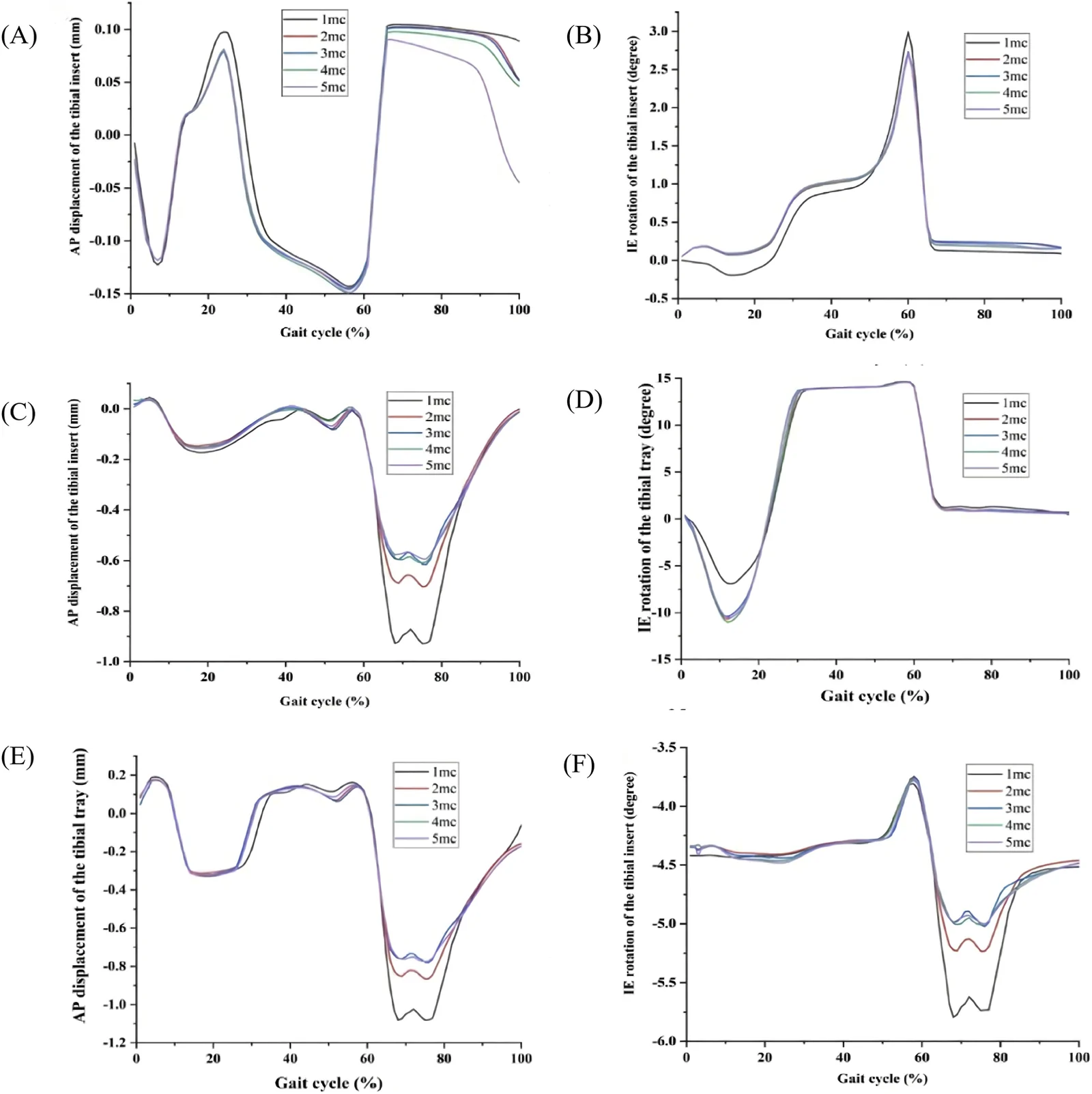
Predicted kinematics from the FE models: **(A,B)** AP translation and IE rotation of the tibial insert of the dual-axis prosthesis; **(C,D)** AP translation and IE rotation of the tibial tray of the ball-and-socket prosthesis; **(E,F)** AP translation and IE rotation of the tibial insert of the ball-and-socket prosthesis.

For the ball-and-socket prosthesis, the tibial tray exhibited a substantially larger AP displacement, ranging from 0 to approximately −1.27 mm (Figure 5C). Posterior translation occurred during the stance phase and gradually recovered toward a neutral position during the transition from stance to swing. The tibial insert demonstrated a similar AP motion pattern with a slightly smaller displacement magnitude of approximately 0.97 mm (Figure 5E). Apart from clearance-related motion, no obvious relative AP translation was observed between the femoral and tibial components.

More pronounced differences were observed in IE rotational kinematics. The tibial tray of the ball-and-socket prosthesis exhibited substantially greater rotational motion than the dual-axis prosthesis, with peak internal rotation increasing from approximately 21.6° at 1 million cycles to 25.6° at 5 million cycles (Figure5D). The tibial tray maintained a relatively large IE rotational excursion throughout most of the stance phase, followed by a rapid reduction during the transition to the swing phase. In contrast, the tibial insert exhibited only limited rotational variation, with its peak-to-peak IE rotational excursion remaining below 3° throughout the gait cycle (Figure 5F). The peak-to-peak excursion decreased from approximately 2.0° at 1 million cycles to 1.2° at 5 million cycles, while the overall rotational pattern remained largely unchanged.

### 3.2 Contact pressure

The contact pressure distributions of the major polyethylene components are shown in Figure 6. In the dual-axis rotating hinge prosthesis, contact was primarily located on the posterior region of the tibial insert during the stance phase (Figure 6A). As the gait cycle progressed, the contact region shifted posteriorly and became more concentrated. Both the medial and lateral sleeves participated in load transmission, with the lateral sleeve exhibiting the highest contact pressure and the most pronounced edge loading. Despite localized stress concentrations, contact remained continuous throughout the gait cycle. The superior surface of the polyethylene insert articulated against the corresponding femoral component.

**FIGURE 6:**
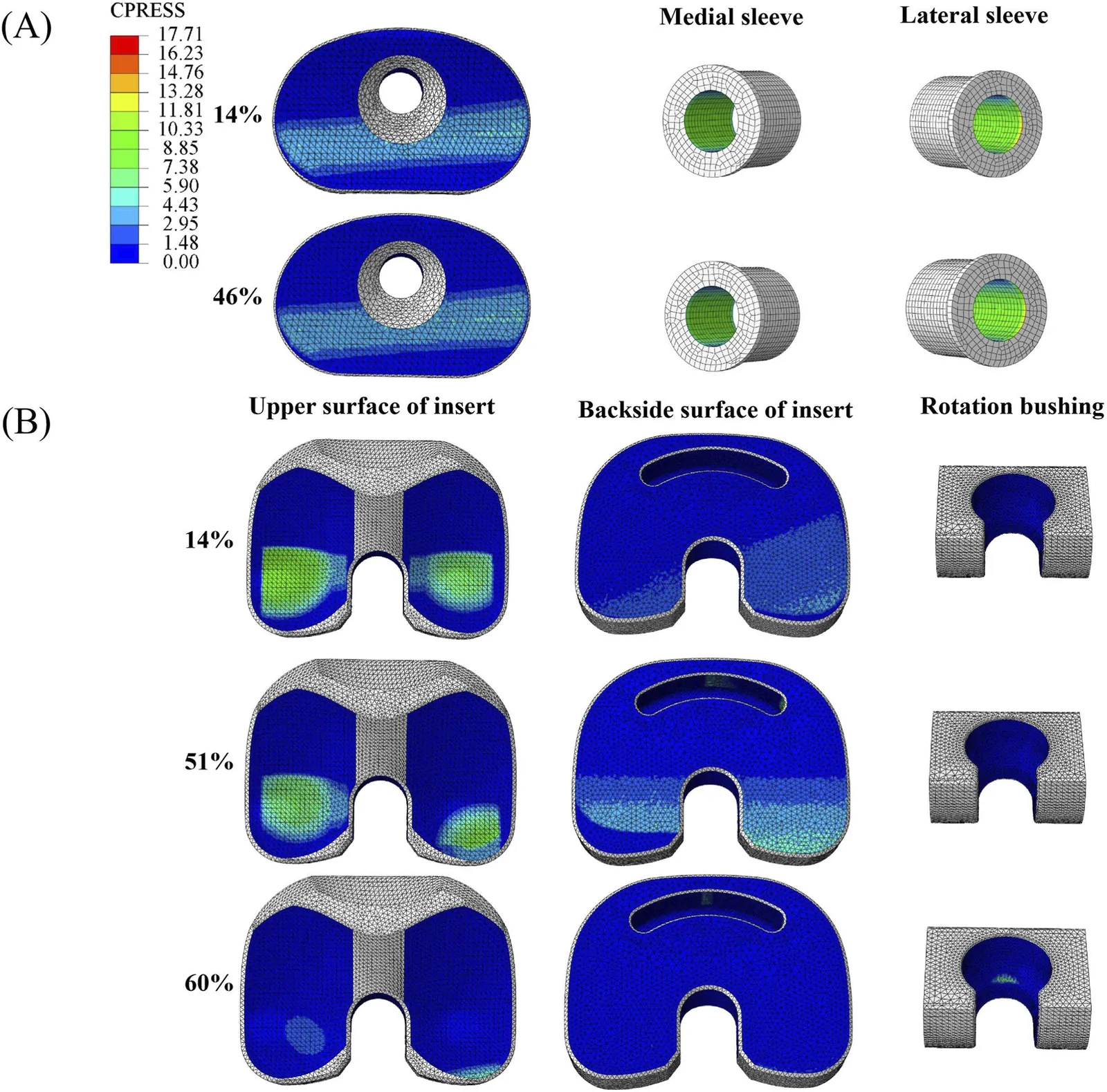
Predicted contact pressure distribution of the two prostheses: **(A)** insert and sleeves of the dual-axis prosthesis; **(B)** upper and analyzed backside surfaces of the insert and rotating bushing of the ball-and-socket prosthesis.

For the ball-and-socket prosthesis, the primary load-bearing contact occurred on the superior surface of the polyethylene insert, whereas substantially lower contact pressures were observed on the groove surface and rotating bushing (Figure 6B). Contact pressures were higher in the inner region of the insert than in the outer region. The contact region gradually migrated posteriorly during the stance phase, while the overall contact pressure distribution remained relatively stable. Continuous contact was observed between the backside surface of the insert and the corresponding metallic component, whereas the rotating bushing contacted the spherical portion of the tibial tray only intermittently.

The maximum contact pressure of the dual-axis prosthesis occurs at the 46% loading cycle phase, while the peak contact pressure of the ball-and-socket prosthesis appears at the 14% loading cycle phase.

Quantitative wear-related parameters are summarized in Table 2. Values represent node-based mean parameters over the analyzed contact surfaces during the simulated gait cycle. The average sliding distance of the dual-axis insert was 0.89 mm, compared with 23.49 mm on the articular surface of the ball-and-socket insert. The average contact area of the dual-axis insert was 575.93 mm^2^, whereas the corresponding articular surface of the ball-and-socket insert measured 345.61 mm^2^. The backside surface of the ball-and-socket insert exhibited the largest average contact area (816.02 mm^2^), while the rotating bushing showed the smallest contact area (17.38 mm^2^). In addition, cross-shear ratios remained close to zero in the dual-axis prosthesis, whereas higher values were observed on the articular surfaces of the ball-and-socket insert.

**TABLE 2:** Wear-related parameters of the two prostheses.

Parameter	Dual-axis prosthesis			Ball-and-socket prosthesis			
	Insert	Medial sleeve	Lateral sleeve	Articular surface of insert	Back side surface of insert	Insert groove	Rotating bushing
Average sliding distance (mm)	0.89	5.43	5.67	23.49	8.33	3.98	1.42
Average contact area (mm^2^)	575.93	416.88	416.88	345.61	816.02	67.01	17.38
Average CS	0.0003	0	0	0.0013	—	—	0.0002

Values represent node-based mean parameters over the analyzed contact surfaces during the simulated gait cycle.

### 3.3 Wear volume

The relationship between accumulated polyethylene wear and the number of gait cycles is shown in Figure 7. After five million cycles, the total wear of the dual-axis rotating hinge prosthesis reached 8.91 mm^3^, corresponding to a wear rate of 1.78 mm^3^/million cycles. For the ball-and-socket prosthesis, the reported total wear of the articular surfae and tthe rotating bushing reached 50.31 mm^3^, corresponding to a wear rate of 10.06 mm^3^/million cycles.

**FIGURE 7:**
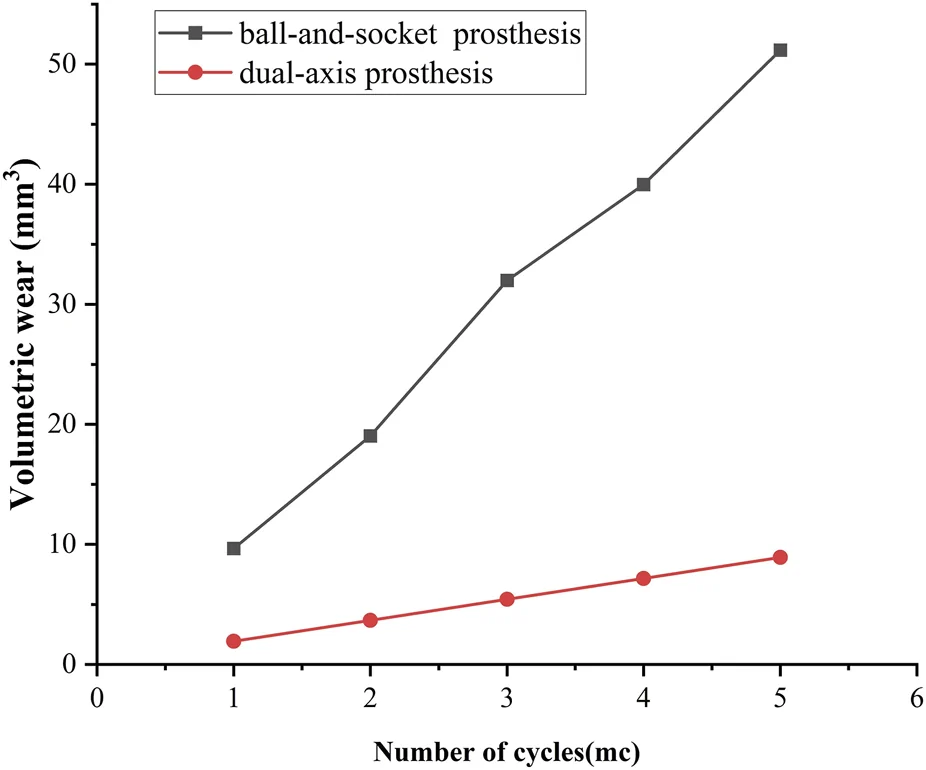
Rlationship between accumulated wear volume and number of cycles for the two prostheses.

Component-specific wear data are summarized in Table 3. Wear increased progressively with the number of simulated gait cycles for all polyethylene components. In the dual-axis prosthesis, the tibial insert showed the greatest wear, increasing from 0.72 mm^3^ at 1 million cycles to 3.65 mm^3^ at 5 million cycles. The medial and lateral sleeves exhibited lower wear throughout the simulation.

**TABLE 3:** Accumulated polyethylene wear volume at each simulated cycle interval for different components of the two prostheses. For the ball-and-socket prosthesis, the reported values refer to the analyzed bearing side and its associated constraint interfaces.

Prosthesis type	Component	Accumulated wear volume (mm^3^)				
		1 million cycles	2 million cycles	3 million cycles	4 million cycles	5 million cycles
Dual-axis prosthesis	Insert	0.72	1.44	2.17	2.90	3.65
	Medial sleeve	0.59	1.09	1.59	2.08	2.57
	Lateral sleeve	0.62	1.14	1.67	2.18	2.69
Ball-and-socket prosthesis	Articular surface of insert	9.42	18.57	31.32	39.11	50.12
	Rotating bushing	0.04	0.09	0.13	0.16	0.19

In the ball-and-socket prosthesis, most wear occurred on the articular surface of the polyethylene insert. Insert wear increased from 9.42 mm^3^ at 1 million cycles to 50.12 mm^3^ at 5 million cycles. In contrast, wear of the rotating bushing remained low and did not exceed 1 mm^3^ during the simulation period. Across all wear cycles, wear of the analyzed ball-and-socket insert surface exceeded that of the dual-axis insert.

## 4 Discussion

### 4.1 Validation of the FE wear model

To evaluate the reliability of the developed finite element (FE) wear model, the numerical predictions were compared with the results of *in vitro* wear experiments performed on the same prosthetic joint design. In this study, the finite element wear prediction exhibited a moderate deviation from experimental test results. Under identical loading parameters and cyclic conditions, the simulated volumetric wear rate was 10 mm^3^ per million cycles, which was 51.28% higher than the experimental value of 6.61 mm^3^ per million cycles (Zhang et al., 2026). This discrepancy is reasonable and explicable given the essential simplifications adopted in numerical modeling. The FE simulation idealized the articular environment by neglecting time-varying lubrication states, microscale surface roughness evolution, and subtle *in vitro* interference factors that universally exist in physical wear tests. In addition, the numerical model adopted fixed friction and contact parameters throughout the iteration process, whereas the actual experimental wear process involves dynamically changing interfacial characteristics and uncontrollable environmental factors. Despite the quantitative difference, the predicted wear magnitude and evolutionary trend are consistent with experimental observations, which verifies the reliability of the established finite element model.

In addition, the contact pressure distribution obtained from the FE analysis showed that the peak pressure region was primarily located in the central articulating area. This region corresponded closely with the wear scars observed in the experimental specimens, demonstrating spatial agreement between the predicted high-contact-pressure regions and the experimentally observed wear pattern. Similar agreement between computational wear prediction and simulator-based wear evaluation has been reported in previous studies of rotating hinge knee prostheses (Zhang et al., 2026).

Although dedicated experimental validation was not performed for both prosthetic designs investigated in the present study, several additional measures were adopted to support the numerical predictions. Material properties, contact definitions, and loading conditions were established according to previously published studies and international standards. Furthermore, the predicted contact pressure distributions, kinematic characteristics, and polyethylene wear trends were consistent with the expected mechanical behavior of highly constrained knee prostheses and remained within the ranges reported in previous simulator and computational studies (Van De Velde et al., 2008; Grupp et al., 2009; 2013). Collectively, these findings provide preliminary support for the use of the FE framework in comparative evaluation of constrained knee prosthesis designs.

### 4.2 Kinematic behavior

The two prosthetic designs exhibited distinct kinematic patterns during simulated gait, reflecting their different constraint mechanisms. The dual-axis prosthesis restricted anterior–posterior (AP) translation through a defined rotational axis and strong mechanical constraint, resulting in limited AP displacement and a stable motion trajectory. In contrast, the ball-and-socket prosthesis allowed greater adaptive motion of the tibial component under gait loading, leading to more pronounced posterior translation during the stance phase. Although AP motion remained relatively small in both designs, the greater translational freedom of the ball-and-socket prosthesis facilitated broader contact migration and longer sliding distances during gait, which are consistent with the highly constrained nature of rotating hinge knee prostheses (Van De Velde et al., 2008; Grupp et al., 2013; León-Román et al., 2021).

More substantial differences were observed in internal-external (IE) rotational kinematics. In the dual-axis prosthesis, rotational motion primarily occurred between the polyethylene insert and the longitudinal connecting axis, while the fixation mechanism of the insert restricted the overall rotational range. Consequently, IE rotation remained below 1.5°for most of the gait cycle and reached only a transient peak of approximately 3.2°. In contrast, rotational motion in the ball-and-socket prosthesis occurred mainly between the tibial tray and femoral component, supplemented by insert rotation, resulting in substantially greater rotational freedom. During the stance phase, IE rotation reached approximately 17°–25° and gradually increased with progressive wear while maintaining a similar overall motion pattern. Although direct clinical kinematic data for ball-and-socket knee prostheses remain limited, the rotational magnitudes observed in the present study exceeded those reported for most condylar-constrained and rotating hinge designs (León-Román et al., 2021).

The greater rotational freedom provided by the ball-and-socket mechanism represents a fundamentally different kinematic strategy from that of the dual-axis design. By allowing adaptive internal rotation during load bearing, the spherical socket structure may better accommodate physiological rotational demands during gait. However, the increased rotational freedom also promoted broader contact migration and more complex multidirectional motion throughout the gait cycle, which may influence the tribological behavior of the prosthesis (Bali et al., 2016; Kahlenberg et al., 2021). Despite the marked differences in rotational magnitude, both prostheses exhibited a rapid reduction in IE rotation at approximately 60% of the gait cycle, indicating that the transition from late stance to swing phase represented the period of greatest rotational change.

### 4.3 Contact mechanics and wear mechanisms

Both prosthetic designs exhibited relatively low contact pressures due to highly conforming or fully congruent articulating surfaces. For the dual-axis prosthesis, this conformity was present at both the insert-femoral component interface and the internal surfaces of the polyethylene sleeves. In the ball-and-socket prosthesis, large contact areas were observed on both the articular and backside surfaces of the insert. Compared with previously reported static analyses of ball-and-socket prostheses (Zhang et al., 2020), the peak contact pressures observed in the present dynamic simulations were lower, likely due to continuous shifts in the contact location during gait.

Despite the localized contact pressure observed in the sleeve regions of the dual-axis prosthesis, the overall wear of the prosthesis remained relatively low. This behavior may be attributed to the decoupled flexion–extension and internal–external rotational motions, which produced predominantly unidirectional sliding patterns and limited multidirectional sliding at the polyethylene interfaces. Consequently, the predicted cross-shear ratios remained close to zero, thereby reducing polyethylene wear. This finding is consistent with previous pin-on-plate and knee simulator studies demonstrating that unidirectional sliding promotes molecular orientation hardening in UHMWPE and improves wear resistance (Kang et al., 2009).

In contrast, the ball-and-socket prosthesis exhibited substantially greater wear despite demonstrating a larger and more uniformly distributed contact area with relatively lower contact pressure. The greater rotational freedom and translational mobility of the design resulted in longer sliding distances and broader contact migration during gait, promoting multidirectional articulation and elevated cross-shear conditions. Therefore, the wear difference between the two designs was primarily driven by motion-path characteristics rather than by contact pressure magnitude. Retrieval studies of rotating hinge knee prostheses have similarly shown that polyethylene damage is strongly influenced by implant-specific motion mechanisms and articulation pathways (Kahlenberg et al., 2021).

These findings suggest that wear behavior in highly constrained knee prostheses may be governed more strongly by multidirectional motion and cross-shear than by contact pressure alone. Although the ball-and-socket prosthesis exhibited favorable contact pressure distributions and greater conformity, its more complex sliding patterns likely increased polyethylene wear. In contrast, the predominantly unidirectional motion of the dual-axis prosthesis resulted in near-zero cross-shear ratios, which may promote molecular orientation hardening and improve wear resistance (Kang et al., 2009; Zhang et al., 2026).

Although the rotating bushing in the ball-and-socket prosthesis was subjected to both flexion-extension and rotational motion, its contact duration, contact area, and contact pressure remained relatively limited throughout the gait cycle, resulting in minimal wear contribution. Similarly, although continuous contact was observed between the backside of the polyethylene insert and the metallic tibial tray, the reported total wear in this study was restricted to the articular surfae and the rotating bushing This definition should be considered when comparing the present values with whole-implant wear measurements from simulator or retrieval studies.

In the present study, the cumulative polyethylene wear volume exhibited a non-steady growth trend with the increase of loading cycles, rather than an ideal linear increment. This typical nonlinear wear characteristic originates from the dynamic coupling of multiple mechanical behaviors during cyclic wear iteration. On the one hand, progressive material loss on the polyethylene articular surface continuously remodels the local contact geometry, which further alters the *in vitro* kinematic performance and contact pressure distribution of the prosthesis. On the other hand, the time-dependent creep deformation of polyethylene under sustained cyclic loading further modulates the interfacial contact state. Combined with the dynamically varying cross-shear magnitude throughout the wear process, these coupled factors collectively lead to fluctuating wear increments at different cyclic stages.

### 4.4 Clinical implications and design considerations

The two constraint mechanisms produced distinct kinematic patterns during gait. The dual-axis rotating hinge prosthesis exhibited negligible anterior-posterior (AP) translation, whereas the AP translational amplitude of the ball-and-socket prosthesis was approximately 3–5 times greater. Although this AP excursion contributed only modestly to the total sliding distance, it increased the multidirectionality of the sliding pathway and consequently the cross-shear rate. In contrast, the internal-external (IE) excursion of the polyethylene insert relative to the metallic component remained relatively small in both prostheses, suggesting that the greater AP motion of the ball-and-socket prosthesis was an important contributor to its increased cross-shear.

These kinematic differences can be explained by the distinct motion mechanisms of the two designs. In the dual-axis prosthesis, flexion-extension motion occurs primarily at the sleeve-spindle interface rather than at the polyethylene insert-metal interface, thereby limiting sliding on the insert articular surface. The sleeve-spindle articulation is predominantly unidirectional, resulting in near-zero cross-shear. Thus, the dual-axis design effectively decouples multidirectional motions. In contrast, flexion-extension motion in the ball-and-socket prosthesis occurs primarily at the femoral component-insert interface and is accompanied by AP translation and rotational accommodation, producing a longer sliding path, broader contact migration, and greater cross-shear.

Despite this more multidirectional motion pattern, the ball-and-socket mechanism demonstrated several potential contact-mechanical advantages. Its greater internal-external rotational freedom and adaptive anterior-posterior motion provided a more rotationally adaptable kinematic response during gait. In addition, the larger and more uniformly distributed contact area resulted in a relatively stable contact-pressure distribution and reduced localized stress concentrations compared with the dual-axis prosthesis. Similar rotational mechanisms have been incorporated into contemporary rotating hinge designs to redistribute torsional loads while maintaining stability in highly constrained knee reconstructions (Oliver et al., 2025).

Published wear data for rotating hinge knee prostheses remain limited. Previous simulator studies have reported combined wear rates for axle bushings and gliding surfaces ranging from approximately 17.7–40.0 mm^3^/million cycles for different rotating hinge knee designs. In comparison, reported wear rates for less-constrained total knee prostheses range from approximately 0.9–41.0 mm^3^/million cycles, depending on implant design, material properties, and testing conditions (Van De Velde et al., 2008; Grupp et al., 2013; 2017). In the present study, the predicted wear rate of the ball-and-socket prosthesis was within the reported range for rotating hinge and conventional total knee prostheses, whereas the dual-axis prosthesis demonstrated substantially lower wear throughout the simulation period. This difference was consistent with the limited sliding distance and near-zero cross-shear of the dual-axis prosthesis, whereas the longer sliding path and greater cross-shear of the ball-and-socket prosthesis produced greater polyethylene wear.

However, the very low predicted wear of the dual-axis prosthesis should be interpreted cautiously. The present wear model assumes that polyethylene wear is independent of contact pressure, an assumption that remains controversial in the literature (O’Brien et al., 2015). Moreover, although the dual-axis prosthesis showed limited multidirectional sliding and near-zero cross-shear in the present computational model, clinical studies have frequently reported substantial polyethylene sleeve wear in rotating hinge knee prostheses (Lee et al., 2013; Haijie et al., 2018; Schroeder et al., 2020), including burnishing, scratching, abrasive damage, and polyethylene deformation of hinge-bearing components (Kahlenberg et al., 2021). Because the sleeves and spindle accommodate the primary flexion-extension motion and act as important load-bearing structures, these components may experience a more demanding tribological environment than represented by the present model. Edge loading, micro-separation, third-body particles, local impingement, and lubrication conditions may also contribute to clinical sleeve wear. Therefore, the very low predicted wear of the dual-axis prosthesis should not be interpreted as evidence of superior long-term clinical wear resistance, but rather as a consequence of its motion mechanism and the simplified boundary conditions and wear formulation used in this study.

From a clinical perspective, restoration of rotational adaptability may be particularly relevant in complex primary and revision knee arthroplasty, in which constrained prostheses are frequently required to compensate for severe ligament insufficiency, extensive bone loss, or tumor-related reconstruction. Under these conditions, controlled rotational and AP accommodation may help maintain joint stability while reducing excessive constraint transfer to the fixation interface.

Therefore, the greater polyethylene wear predicted for the ball-and-socket prosthesis should not be considered simply as an unfavorable trade-off for increased motion. After further experimental validation and design optimization, the ball-and-socket configuration may represent an alternative reconstructive strategy that balances joint stability, rotational adaptability, contact-mechanical performance, and wear resistance.

Future optimization of ball-and-socket prostheses should focus on reducing excessive multidirectional sliding and contact migration while preserving rotational adaptability. Potential strategies include optimization of spherical conformity, rotational center position, and backside locking mechanisms to achieve a more favorable balance between rotational freedom, contact distribution, cross-shear, and polyethylene wear.

### 4.5 Limitations

Several limitations should be acknowledged. First, the loading and motion profiles defined by ISO 14243 were not specifically developed for highly constrained knee prostheses. Although these standardized conditions enabled comparison between prosthetic designs, patient-specific factors such as gait pattern, soft-tissue balance, and implant alignment may alter rotational motion, contact migration, and multidirectional sliding behavior, thereby influencing polyethylene wear *in vivo*. Therefore, the present results should be interpreted as a standardized comparative assessment rather than a direct prediction of *in vivo* wear. Second, the present wear model assumed that polyethylene wear was independent of contact pressure, although this assumption remains controversial in the literature. Since the present study suggests that wear is governed more strongly by multidirectional motion than contact pressure alone, the relative contributions of these factors require further experimental investigation. Third, only the analyzed bearing side and associated constraint interfaces of the ball-and-socket prosthesis were included in the reported total wear volume; additional symmetric or contralateral bearing surfaces were not included in the reported total, which limits direct extrapolation to whole-implant wear *in vivo*. Fourth, backside wear of the dual-axis prosthesis insert was not considered because of the rigid fixation between the polyethylene insert and tibial tray. Although interface micromotion was expected to be limited, fretting wear and polyethylene debris generation may still occur during long-term clinical use and potentially influence implant longevity (Geringer and Macdonald, 2012; Arnholt et al., 2016; Bali et al., 2016). Fifth, a uniform friction coefficient of 0.04 derived from conventional metal-on-polyethylene articulating surfaces under boundary lubrication was applied to all contact interfaces of both prosthetic models. Although this value is widely accepted for primary bearing surfaces in knee prosthesis wear simulations, the secondary constraint interfaces, including the flexion shaft–bushing and ball–bushing contact pairs, possess different structural geometries, contact mechanics, and lubrication environments. Assigning identical friction coefficients to all interfaces may neglect the interfacial differences in friction behavior and lubrication conditions. Further studies should establish differentiated friction parameters for different contact interfaces based on experimental measurements to improve the accuracy of wear prediction. Sixth, lubrication and fluid-structure interaction effects were not incorporated into the finite element model. Since the *in vitro* experiments were conducted under serum lubrication conditions, the absence of lubrication modeling may influence the predicted contact mechanics, multidirectional sliding behavior, and wear mechanisms. Therefore, further experimental validation remains necessary to improve the clinical relevance of the computational predictions. Finally, this study mainly focuses on the wear performance of knee prostheses; therefore, bone and implant fixation models were not incorporated in the current finite element framework. The material properties and pressure-independent wear model adopted in this work were derived from published literature without targeted calibration and parametric investigation. In addition, parametric sensitivity analysis was not conducted in this study. Furthermore, the experimental validation was only performed for a single prosthesis configuration, which limits the comprehensiveness of model verification.

## 5 Conclusion

This study developed a dynamic finite element framework to compare the kinematics, contact mechanics, and polyethylene wear behavior of dual-axis rotating hinge and ball-and-socket knee prostheses under gait loading conditions. The results demonstrated that prosthetic design substantially influenced joint kinematics and wear behavior in highly constrained knee systems.

Compared with the dual-axis rotating hinge prosthesis, the ball-and-socket prosthesis provided greater internal-external rotational accommodation. However, this increased rotational freedom was associated with longer sliding distances, broader contact migration, and higher cross-shear, resulting in greater polyethylene wear despite relatively low contact pressure.

These findings suggest that the ball-and-socket mechanism may represent a promising alternative design strategy for constrained knee reconstruction, but further experimental validation and design optimization are required to reduce excessive contact migration and cross-shear while preserving rotational adaptability.

## Data Availability

The raw data supporting the conclusions of this article will be made available by the authors, without undue reservation.
