# Supplementary material for: Influence of constraint mechanism on kinematics, contact mechanics, and polyethylene wear in dual-axis rotating hinge and ball-and-socket knee prostheses: a finite element study
Source: Front Bioeng Biotechnol. 2026 Sep 23;14:1942105. doi: 10.3389/fbioe.2026.1942105 (PMC13642287; doi:10.3389/fbioe.2026.1942105)
Supplement: Supplementary file 1 [file DataSheet1.docx]

Supplementary Material

# 1 Supplementary Table

Table 1.Prescribed and constrained degrees of freedom for the dual-axis and ball-and-socket prostheses

|  | Degrees of Freedom | | | | |
| --- | --- | --- | --- | --- | --- |
|  | FE | IE | AA | ML | AP |
| Dual-axis prosthesis | √ | √ | × | × | × |
| Ball-and-sock prosthesis | √ | √ | × | × | × |

*FE:flexion-extension IE:internal–external rotation AA:abduction–adduction

ML:medial–lateral translation AP:anterior–posterior translation

Table 2. Wear-related parameters of the two prostheses. Values represent node-based mean parameters over the analyzed contact surfaces during the simulated gait cycle.

|  | Dual-axis prosthesis | | | Ball-and-socket prosthesis | | | |
| --- | --- | --- | --- | --- | --- | --- | --- |
|  | Insert | Medial sleeve | Lateral sleeve | Articular surface of insert | Back side  Surface of insert | Insert groove | Rotating bushing |
| Average sliding distance (mm) | 0.89 | 5.43 | 5.67 | 23.49 | 8.33 | 3.98 | 1.42 |
| Average contact area (mm^2^) | 575.93 | 416.88 | 416.88 | 345.61 | 816.02 | 67.01 | 17.38 |
| Average CS | 0.0003 | 0 | 0 | 0.0013 | — | — | 0.0002 |

Table 3. Accumulated polyethylene wear volume at each simulated cycle interval for different components of the two prostheses. For the ball-and-socket prosthesis, the reported values refer to the analyzed bearing side and its associated constraint interfaces.

|  |  | Accumulated wear volume (mm^3^) | | | | |
| --- | --- | --- | --- | --- | --- | --- |
|  |  | 1 million cycles | 2 million cycles | 3 million cycles | 4 million cycles | 5 million cycles |
| Dual-axis prosthesis | Insert | 0.72 | 1.44 | 2.17 | 2.90 | 3.65 |
|  | Medial sleeve | 0.59 | 1.09 | 1.59 | 2.08 | 2.57 |
|  | Lateral sleeve | 0.62 | 1.14 | 1.67 | 2.18 | 2.69 |
| Ball-and-socket prosthesis | Articular surface of insert | 9.42 | 18.57 | 31.32 | 39.11 | 50.12 |
|  | Rotating bushing | 0.04 | 0.09 | 0.13 | 0.16 | 0.19 |

# 2 Supplementary Figures

## 2.1 Supplementary Figures

**Figure 1.** The CAD model and component parts of the two prostheses: (A) dual-axis prosthesis; (B) ball-and-socket prosthesis.

**Figure 2.** Illustration of FE models and motion of the two prostheses: (A) the dual-axis prosthesis; (B) the ball-and-socket prosthesis.

**Figure 3.** real stress‑strain curve of The UHMWPE

**Figure 4.** Flowchart for wear simulation of hinged knee prosthesis

Figure 5. Predicted kinematics from the FE models: (A-B) AP translation and IE rotation of the tibial insert of the dual-axis prosthesis; (C-D) AP translation and IE rotation of the tibial tray of the ball-and-socket prosthesis; (E-F) AP translation and IE rotation of the tibial insert of the ball-and-socket prosthesis.

Figure 6. Predicted contact pressure distribution of the two prostheses: (A) insert and sleeves of the dual-axis prosthesis; (B) upper and analyzed backside surfaces of the insert and rotating bushing of the ball-and-socket prosthesis.

Figure 7. Rlationship between accumulated wear volume and number of cycles for the two prostheses.
